# *SERPINC1* c.1247dupC: a novel *SERPINC1* gene mutation associated with familial thrombosis results in a secretion defect and quantitative antithrombin deficiency

**DOI:** 10.1186/s12959-024-00589-5

**Published:** 2024-02-12

**Authors:** Maximilian Ruf, Sarah Cunningham, Alexandra Wandersee, Regine Brox, Susanne Achenbach, Julian Strobel, Holger Hackstein, Sabine Schneider

**Affiliations:** Department of Transfusion Medicine and Hemostaseology, Friedrich-Alexander-University Erlangen-Nürnberg (FAU), University Hospital Erlangen, Krankenhausstr. 12, 91054 Erlangen, Germany

**Keywords:** Hereditary antithrombin deficiency, Frameshift mutation, Secretion defect, Thrombosis

## Abstract

**Background:**

Antithrombin (AT) is an important anticoagulant in hemostasis. We describe here the characterization of a novel AT mutation associated with clinically relevant thrombosis. A pair of sisters with confirmed type I AT protein deficiency was genetically analyzed on suspicion of an inherited *SERPINC1* mutation. A frameshift mutation, c.1247dupC, was identified and the effect of this mutation was examined on the cellular and molecular level.

**Methods:**

Plasmids for the expression of wild-type (WT) and mutated *SERPINC1* coding sequence (CDS) fused to green fluorescent protein (*GFP*) or hemagglutinin (*HA*) tag were transfected into HEK293T cells. Subcellular localization and secretion of the respective fusion proteins were analyzed by confocal laser scanning microscopy and Western blot.

**Results:**

The c.1247dupC mutation results in a frameshift in the CDS of the *SERPINC1* gene and a subsequently altered amino acid sequence (p.Ser417LysfsTer48). This alteration affects the C-terminus of the AT antigen and results in impaired secretion as confirmed by GFP- and HA-tagged mutant AT analyzed in HEK293T cells.

**Conclusion:**

The p.Ser417LysfsTer48 mutation leads to impaired secretion, thus resulting in a quantitative AT deficiency. This is in line with the type I AT deficiency observed in the patients.

**Supplementary Information:**

The online version contains supplementary material available at 10.1186/s12959-024-00589-5.

## Background

AT is a single-chain glycoprotein (464 amino acids containing a 32 amino acid N-terminal signal peptide) and is secreted as a 432 amino acid protein by hepatocytes into the blood stream [1, 2]. AT has two major binding sites for interaction partners: the heparin-binding site at the N-terminus and the reactive site at the C-terminus. AT acts as a serine protease inhibitor (SERPIN) by binding covalently with the Arg425-Ser426 peptide bond in its reactive center loop to the active site of serine proteases in the blood. This way AT inactivates, next to other serine proteases, coagulation factors IIa (thrombin), IXa, Xa, XIa and XIIa [1, 2].

There are two types of AT deficiencies. Type I deficiency is characterized by concordant reduction of protein level and activity, while type II deficiency is defined by normal protein levels but a reduced functionality. Type II deficiency of AT can be subdivided into three groups depending on the affected domain of the AT protein. In type IIa, mutations affect the reactive site, in type IIb, mutations affect the heparin-binding site and in type IIc, mutations show different defects in other parts of the protein [3]. Independent of its classification, AT deficiency, with a prevalence of 0.1–0.3% within the Caucasian population, poses a major risk for thromboembolic events [4, 5].

## Methods

### Samples

The here discussed patients were two sisters who were diagnosed with AT deficiency after multiple thromboembolic events. Patient 1, a 64-year-old woman, suffered from a bilateral deep vein thrombosis (DVT) as a teenager with recurrence after a temporary change in her anticoagulation treatment from phenprocoumon to acetylsalicylic acid. The treatment regimen was switched back to phenprocoumon after the DVT. Her sister (patient 2), a 65-year-old woman, had multiple DVT events in her right lower leg and pulmonary embolism as a teenager. The patient also developed a DVT in her right leg at the age of 27 during pregnancy eight weeks before delivery. The intake of an oral contraceptive during the time of their first thrombotic events was postulated as a possible triggering factor. In addition, both patients have been diagnosed with a heterozygous factor V Leiden (FVL) mutation. According to the family history, the patients´ father was diagnosed with AT deficiency. No statement could be made from the patients as to whether their parents suffered from thromboembolic events. Additionally, the patients could not provide any information regarding the heredity of the FVL mutation within their family.

Blood samples from healthy blood donors of the blood donation center of the Clinical Department of Transfusion Medicine and Hemostaseology in Erlangen served as control material within the study. The study was conducted in accordance with the Declaration of Helsinki, and approved by the Ethics Committee of the Friedrich-Alexander-University Erlangen-Nürnberg (FAU) (#477_20 B, #357_19 B, #343_18 B). Written informed consent was obtained from all participants of the study.

### Determination of AT parameters

AT activity and AT antigen levels were analyzed with a STA Max instrument (Diagnostica Stago S.A.S., Asnières-sur-Seine, France), using the STACHROM AT III Kit for activity and the LIATEST AT III kit for antigen levels (Diagnostica Stago S.A.S., Asnières-sur-Seine, France).

### DNA isolation

Genomic DNA was automatically isolated from whole blood and quantified using commercial kits (NucleoSpin® 8 Blood Core Kit, Macherey Nagel, Düren, Germany for isolation, QuantiFluor® dsDNA System, Promega, Madison, WI, USA for quantification) and a Hamilton Microlab Starlet robot (Reno, NV, USA) and microplate fluorometer (Berthold, Bad Wildbad, Germany).

### Next generation sequencing

The CDS (according to transcript NM_000488.3) and adjacent intron regions of the *SERPINC1* gene were sequenced with an Illumina amplicon-based gene panel (AmpliSeq™ Custom DNA Panel for Illumina®) on a MiSeq machine (Illumina®, San Diego, CA, USA) in accordance to the manufacturer’s instructions. The custom DNA panel enabled CDS and adjacent intron regions sequencing of the following genes: *F7*, *F9*, *F13A1*, *F13B*, *F11*, *FGA*, *FGB*, *FGG*, *PROC*, *PROS1*, *SERPINC1* and *VWF* [6].

Next generation sequencing data was thereafter analyzed with the SeqNext Software version 5.2.0 (JSI Medical Systems, Ettenheim, Germany).

### Database submission

The c.1274dupC frameshift mutation was submitted to ClinVar (www.ncbi.nlm.nih.gov/clinvar; accession SCV002520629).

### Quantitative mutation screening

To gage the prevalence of the c.1247dupC frameshift mutation within the general population, 358 healthy individuals were screened with a sequence-specific primer polymerase chain reaction (SSP-PCR) as described before [6]. Oligonucleotides were obtained from Merck (Darmstadt, Germany). Primer combinations consisted of the following: SERPINC1g+13006f (5’-AGTACCTTACATTCTCTGCATGA-3’) and SERPINC1g+13243r-WT (5’-CAATCACAACAGCGGTACTTGC-3’) for the *SERPINC1* WT allele (resulting in a 238 bp fragment) and SERPINC1g+13006f and SERPINC1g+13243r-SNP (5’-CAATCACAACAGCGGTACTTGG-3’) for detecting the *SERPINC1* c.1247dupC frameshift mutation (resulting in a 239 bp fragment). The co-amplification of the human growth hormone gene (*GH1*) with primers GH1g+334f (5’-TGCCTTCCCAACCATTCCCTTA-3’) and GH1g+768r (5’-CCACTCACGGATTTCTGTTGTGTTTC-3’), resulting in a 434 bp fragment, served as an internal control.

### Construct generation

Plasmids for HEK293T cell transfection were created using Gibson Assembly [7], according to the manufacturer’s instruction manual (Gibson Assembly® Master Mix/Gibson Assembly® Cloning Kit NEB, New England Biolabs, Ipswich, MA, USA). Oligonucleotides for Gibson Assembly were designed using NEBuilder Assembly Tool (https://nebuilder.neb.com/#!/) and were manufactured by Merck (Darmstadt, Germany). Vector pcDNA3.1( +)/Puro/FA/GFP-LL5BIP [kindly provided from Tomasz Prószyński (Addgene plasmid # 112829; http://n2t.net/addgene:112829; RRID:Addgene_112829)] was used for the expression of the *GFP* fusion. For plasmid pMR01 (*SERPINC1*-*GFP*) insert sequences and vector fragments with complementary overhangs were amplified by PCR using primers (and templates) as follows:

- *SERPINC1* CDS fragment: MR01-SERPINC1_fwd (5’-GTACCGAGCTCGGATCCATGTATTCCAATGTGATAGGAACTGTAACCTCTG-3’), MR01-SERPINC1_rev (5’-CACCATACCGCTACCGCCGCCGCTGCCACCCTTAACACAAGGGTTGGCTACTCTGC-3’) (human antithrombin III cDNA ORF Clone, obtained from SinoBiological, Beijing, China)
- GFP CDS fragment: MR01-GFP_fwd (5’-GTTAAGGGTGGCAGCGGCGGCGGTAGCGGTATGGTGAGCAAGGGCGAGGAG-3’), MR01-GFP_rev (5’-GCACAGTCGAGGCTGATCACTTGTACAGCTCGTCCATGCCG-3’) (pcDNA3.1(+)/Puro/FA/GFP-LL5BIP)
- vector pcDNA3.1 backbone: pcDNA3.1_fwd (5’-GGACGAGCTGTACAAGTGATCAGCCTCGACTGTGCCTTCTAG-3’), pcDNA3.1_rev (5’-CTATCACATTGGAATACATGGATCCGAGCTCGGTACCAAG-3’ (pcDNA3.1(+)/Puro/FA/GFP-LL5BIP).

For plasmid pMR02 (*SERPINC1*_c.1247dupC_-GFP) the c.1247dupC mutation was introduced by site-directed mutagenesis using the Q5 Site-Directed Mutagenesis Kit (New England Biolabs, Ipswich, MA, USA) [8], with the primer combination M1-pBWCS2_DupC_fwd (5’-GCAGCTGCCAAGTACCGCTGTTGTG-3’) and M1-pBWCS2_DupC_rev (5’-CGGTACTTGGCAGCTGCTTCACTG-3’). To deal with the resulting frameshift that also affects the CDS of *GFP*, *SERPINC1* c.1247dupC CDS was amplified with oligonucleotides MR01-SERPINC1_fwd (5’-GTACCGAGCTCGGATCCATGTATTCCAATGTGATAGGAACTGTAACCTCTG-3’) and MR02-ohne Stop_rev (5’-GCTACCGCCGCCGCTGCCACCACACAAGGGTTGGCTACTCTGCC-3’) followed by the assembly into the pMR01 backbone (obtained with primer combination MR02-GGSG-EGFP_fwd: 5’-GGTGGCAGCGGCGG-3’ and MR01-pcDNA3.1_rev: 5’-CTATCACATTGGAATACATGGATCCGAGCTCGGTACCAAG-3’). During the PCR amplification for the Gibson Assembly a 2 × GGSG linker was introduced in front of the GFP in both pMR01 and pMR02.

The HA tag fusion vector was generated by digesting pcDNA3.1( +)/Puro/FA/GFP-LL5BIP with HindIII and XbaI to remove the GFP-LL5BIP cassette. Oligonucleotides encoding a 2 × GGSG linker and a 3 × HA tag (HindIII-GGSG-3xHA-Tag-XbaI_fwd: 5’-CTAGATCAAGCATAGTCAGGTACGTCATAAGGGTAAGATCCAGCATAGTCAGGTACGTCATAAGGGTAAGATCCAGCATAGTCAGGTACGTCATAAGGGTAACCGCTACCGCCA-3’ and HindIII-GGSG-3xHA-Tag-XbaI_rev: 5’-CTAGATCAAGCATAGTCAGGTACGTCATAAGGGTAAGATCCAGCATAGTCAGGTACGTCATAAGGGTAAGATCCAGCATAGTCAGGTACGTCATAAGGGTAACCGCTACCGCCA-3’) were annealed, leading to a double-stranded DNA fragment with HindIII and XbaI overhangs. This fragment was ligated into the HindIII/XbaI digested pcDNA3.1 backbone, resulting in the HA tag expression vector pBWCS3.

Insert and vector fragments for plasmid pMR03 (*SERPINC1*-*HA*) were amplified using primers (and template) as follows:

- *SERPINC1* CDS fragment: MR03_SERPINC-3xHA_fwd (5’- GCTAGCGTTTAAACTTAAGCTTATGTATTCCAATGTGATAGGAACTGTAACCTCTG-3’), pMR03_rev (5’-CATAAGGGTAACCGCTACCGCCGCCGCTGCCACCCTTAACACAAGGGTTGGCTACTCTGC-3’) (pMR01)
- vector pBWCS3 backbone: pBWCS3-GGSG-3xHA_fwd (5’-GGCGGTAGCGGTTACCCTTATG-3’), pBWCS3-GGSG-3xHA_rev (5’-AAGCTTAAGTTTAAACGCTAGCCAGCTTG-3’) (pBWCS3).

For plasmid pMR04 (*SERPINC1*_c.1247dupC_-HA) the insert and vector fragments were amplified by PCR using the following primers (and template):

- *SERPINC1* c.1247dupC: MR03_SERPINC-3xHA_fwd (5’-GCTAGCGTTTAAACTTAAGCTTATGTATTCCAATGTGATAGGAACTGTAACCTCTG-3’), pMR04_rev (5’-CATAAGGGTAACCGCTACCGCCGCCGCTGCC ACCACACAAGGGTTGGCTACTCTGC-3’) (pMR02)
- vector pBWCS3 backbone: pBWCS3-GGSG-3xHA_fwd (5’-GGCGGTAGCGGTTACCCTTATG-3’), pBWCS3-GGSG-3xHA_rev (5’-AAGCTTAAGTTTAAACGCTAGCCAGCTTG-3’) (pBWCS3).

Plasmid sequences were validated and confirmed by Sanger sequencing (Eurofins Genomics Germany GmbH, Ebersberg, Germany).

### HEK293T clone generation

HEK293T cells (ACC 635, DSMZ, Braunschweig, Germany) were grown in DMEM (#D6429, Sigma-Aldrich, Taufkirchen, Germany) supplemented with 10% FCS (anprotec, Bruckberg, Germany), 1% GlutaMAX (Thermo Fisher Scientific, Waltham, MA, USA), 1% Penicillin–Streptomycin (Sigma-Aldrich, Taufkirchen, Germany) at 37°C and 5% CO_2_ in a humidified incubator. Cells were transfected with *Trans*IT®-293 Reagent (Mirus Bio LLC, Madison, WI, USA) following the manufacturer´s recommendations. After 24 h the medium was replaced with fresh medium containing 5 µg/ml Puromycin (Carl Roth, Karlsruhe) to select the transfected cells. Clones were picked after 1–2 weeks and analyzed for the presence of the respective tag by flow cytometry (CytoFLEX S, Beckman Coulter, Brea, CA, USA). HEK293T cells transfected with pMR01 and pMR02 were analyzed for direct *GFP* expression while *HA*-tag expression of HEK293T cells transfected with pMR03 and pMR04 was assessed by intracellular staining in accordance to the manufacturer’s protocol (HA Tag Monoclonal antibody Dy550, #26183D550, Life Technologies, Carlsbad, CA, USA; eBioscience™ FOXP3-staining kit, #00–5523-00, Thermo Fisher Scientific, Waltham, MA, USA). Up to six cell lines were picked for each plasmid transfection and used for subsequent confocal laser scanning microscopy and Western blot.

Prior to microscopic analysis, HEK293T cells expressing plasmid pMR01 or pMR02 were transfected prior to imaging with the ER-Tracker™ Red (glibenclamide BODIPY® TR) (Thermo Fisher Scientific, Waltham, MA, USA) for 30 min following the manufacturer´s recommendations.

### Confocal laser scanning microscopy

A Leica TCS SP8 confocal laser scanning microscope (Leica, Wetzlar, Germany) was used for imaging with 488 nm (GFP) and 552 nm (RFP) laser light for excitation. GFP fluorescence was detected in a window ranging from 495–520 nm and RFP fluorescence in a window ranging from 570–595 nm. Data processing was performed with the Leica Application Suite X software version 2.0.1.14392 (Leica, Wetzlar, Germany).

### Western blotting

In order to quantify protein content and secretion, respective HEK293T clones were cultured at 2 × 10^6^–1 × 10^7^ cells/ml in serum-free culture medium (#14571C, EX-CELL®, Merck, Darmstadt, Germany) without antibiotics for 48 h. Cells were subsequently harvested by resuspension and centrifuged at 300 g for 5 min. Supernatants were collected, mixed with the cOmplete™ Mini protease inhibitor-cocktail (Merck, Darmstadt, Germany) and subsequently stored at -20°C. The cell pellets were lysed with Radioimmunoprecipitation Assays buffer (RIPA: 50 mM Tris–HCl, pH 8.0, 150 mM NaCl, 0.5% sodium deoxycholate, 0.1% Triton X-100 and 0.1% SDS) and the cOmplete™ Mini protease inhibitor-cocktail (Merck, Darmstadt, Germany) for 30 min on ice. Samples were subsequently centrifuged at 11,000 g for 10 min at 4°C. The ensuing cell lysate supernatants were transferred into a fresh tube and stored at -20°C until further use.

Protein concentrations were determined with a Bradford assay (Roti®-Nanoquant #K880.1, Carl Roth, Karlsruhe, Germany) according to the manufacturer’s protocol. The measurement was performed on a Fluostar Omega (BMG Labtech, Ortenberg, Germany) at 590/450 nm.

SDS-PAGE was performed with 40 µg protein from cell lysates and 5 µg protein from supernatant reduced in 4 × Laemmli-buffer (Roti®-Load1, #K929.1, Carl Roth, Karlsruhe, Germany). Samples were denaturized at 95°C for 5 min and subsequently loaded onto 4–15% Mini-PROTEAN® TGX™ Precast Protein Gels (Bio-Rad, Hercules, CA, USA). Protein bands were seperated at 100 V for 1 h with Western Blot Running Buffer (25 mM TRIS Base, 192 mM Glycin, 0.1% SDS; pH 8.3) using the Mini-PROTEAN® Tetra Vertical Electrophoresis System (Bio-Rad, Hercules, CA, USA). After separation, gels were blotted onto a nitrocellulose membrane (#GE10600003, Amersham™ Protran® Premium, Sigma-Aldrich, Taufkirchen, Germany) at 100 V for 1 h (Mini Trans-Blot Module, Bio-Rad, Hercules, CA, USA).

Membranes were washed in Tris-buffered saline with Tween solution (TBST: 20 mM TRIS, pH 7.5, 150 mM NaCl, 0.1% Tween-20) and blocked with TBST containing 5% milk powder (Carl Roth, Karlsruhe, Germany) for 1 h at room temperature. Immunostaining of GFP fusion proteins of cell lysate and supernatant samples was done with GFP Polyclonal Antibody, DyLight™ 800 (1:10,000 dilution; #600–145-215, Thermo Fisher Scientific, Waltham, MA, USA). HA tagged proteins within cell lysate and supernatant samples were immunostained with HA Tag Monoclonal Antibody (2–2.2.14), DyLight™ 550 (1:1000 dilution; #26183D550, Thermo Fisher Scientific, Waltham, MA, USA). GAPDH Loading Control Monoclonal Antibody (GA1R), DyLight™ 680, Thermo Fisher Scientific, Waltham, MA, USA, was used to normalize the Western blot. The blots were stained at 4°C overnight and washed the next day three times for 10 min in TBST. A ChemiDoc Imaging System, Bio-Rad (Hercules, CA, USA) was used for imaging.

GAPDH loading control monoclonal antibody was used for normalization of protein amount from cell lysates. Supernatants were normalized to the total protein content within the stain-free gels. A ChemiDoc MP Imaging System (Bio-Rad, Hercules, CA, USA) was used for imaging.

### Statistical analysis

Basic statistics (mean values and standard deviations) were performed using Microsoft Excel 2016 (Office Professional Plus 2016; Microsoft, Redmond, WA, USA) and visualized using GraphPad Prism (version 9.51). Results are shown as mean values ± standard deviation. One-way ANOVA followed by Bonferroni and Holm multiple comparison (all pairs simultaneously compared) (https://astatsa.com/) was used to test for differences between cell lines.

### In silico prediction of protein structure

Tertiary structure of WT and mutated AT was predicted using Contact-guided Iterative Threading ASSEmbly Refinement (C-I-TASSER) [9]. Furthermore, iCn3D was used to display the calculated models [10, 11].

## Results

### Identification of *SERPINC1* mutation c.1247dupC (p.Ser417LysfsTer48)

Patient 1 presented reduced AT antigen levels and reduced AT activity with 44% and 57%, respectively, with normal ranges from 80–120% for both.

Patient 2 also presented reduced AT antigen level of 48% and a reduced AT activity of 60%.

**Fig. 1:**
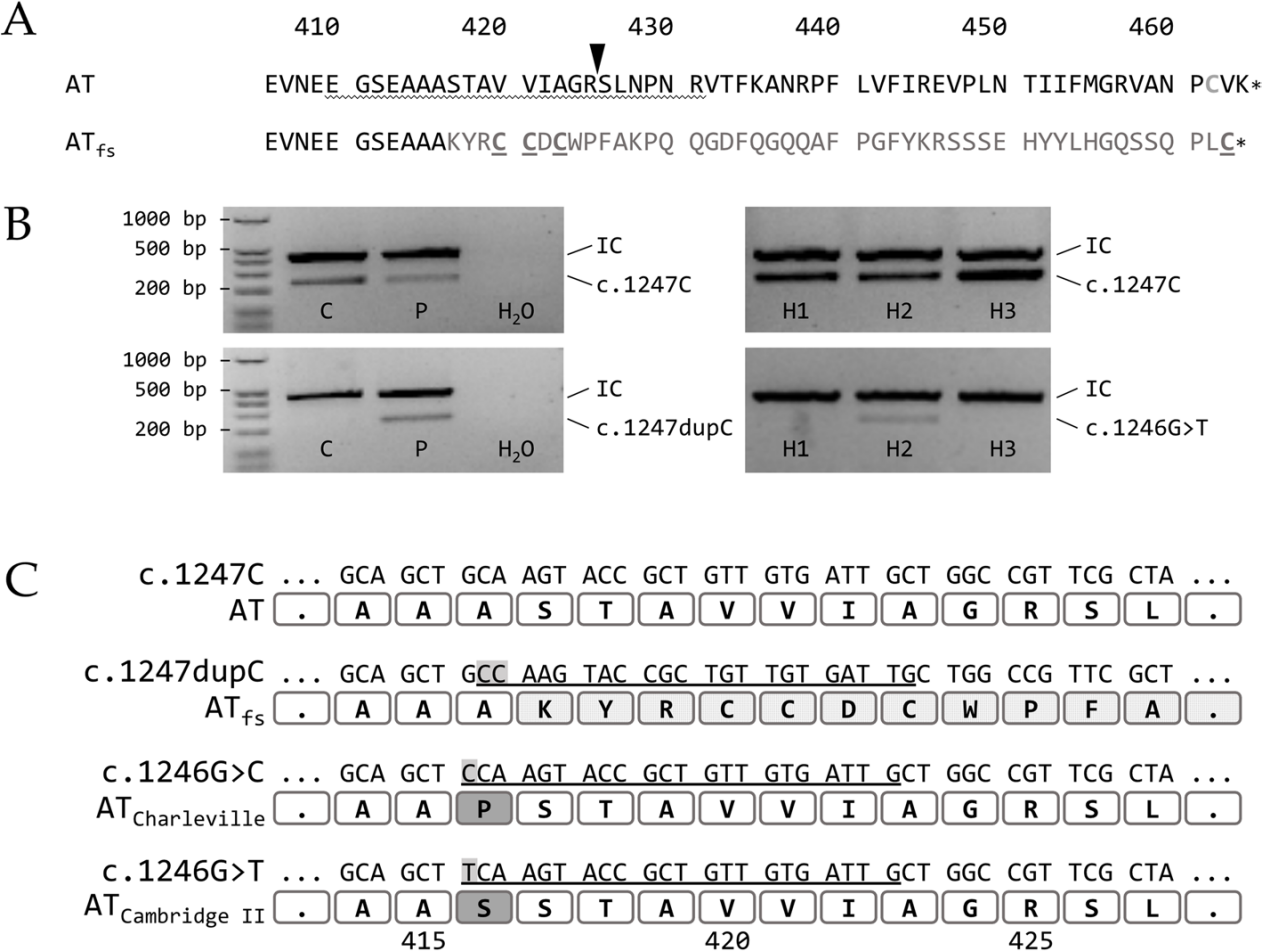
The *SERPINC1* + 1 frameshift mutation c.1247dupC results in an altered AT C-terminus (p.Ser417LysfsTer48). **A** Comparison of the C-terminal amino acid sequence of WT (AT) and mutant AT (AT_fs_) with amino acid sequence change p.Ser417LysfsTer48 given in gray. The reactive center loop is underlined, the arrowhead indicates the position of the reactive Arg-Ser bond. Cys462, involved in a disulfide bond in AT, is shown in light gray. New Cys residues introduced by the frameshift mutation in AT_fs_ are underlined. **B** Exemplary results of SSP-PCR screening for the *SERPINC1* c.1247dupC mutation in 358 healthy individuals. Upper row: screening for the WT allele using genomic DNA of a healthy individual (C) and a patient described (P) as controls and three out of 358 healthy individuals (H1, H2, H3). The PCR result for the *SERPINC1* WT allele is represented by a 238 bp fragment (c.1247C). Lower row: screening for the c.1247dupC allele. Genomic DNA of a healthy individual (C) and a patient (P) were used as controls. The primer combination for detection of the c.1247dupC allele (239 bp) also amplified the AT_Cambridge II_ allele (c.1246G>T) [12], which was found in three out of 358 healthy individuals (H2 shown as example). H_2_O, water control; IC, internal PCR control (434 bp fragment from *GH1* gene). **C** Comparison of partial DNA sequences of *SERPINC1* WT (c.1247C), the described frameshift mutation (c.1247dupC) and mutations AT_Charleville_ (c.1246G>C) [13] and AT_Cambridge II_ (c.1246G>T) [12], which could also possibly be detected. The respectively encoded amino acid sequence is given underneath for orientation. Underlined nucleotides indicate the position of the reverse primer used for detection of the mutant allele; nucleotides highlighted by a gray background indicate the respective mutation. Amino acids highlighted by a dotted background indicate the p.Ser417LysfsTer48 mutation. The Ala416Pro and Ala416Ser exchanges, encoded by the AT_Charleville_ mutation c.1246G>C [13] and AT_Cambridge II_ mutation c.1246G>T [12], respectively, are highlighted by a gray background

Since a hereditary AT deficiency was suspected, genetic analysis was performed for both patients. The analysis showed the identical heterozygous insertion (c.1247dupC) within exon 7 (according to gene model NM_000488.3) for both patients. This mutation leads to a frameshift within the AT CDS, resulting in a switch from Ser417 to Lys and altering the subsequent 46 amino acids (p.Ser417LysfsTer48; hereafter indicated as AT_fs_) (Fig. 1A). Coincidentally, the C-terminus of AT_fs_ differs by only one amino acid in length, although the amino acid sequence is completely different.

Three hundred and fifty-eight healthy individuals were screened for the c.1247dupC mutation via SSP-PCR in order to assess the frequency within the general population. Three individuals tested positive within the healthy cohort using the primer combination to amplify the c.1247dupC allele (exemplarily shown for one individual, H2, in Fig. 1B).

There are two known mutations at nucleotide c.1246 that could possibly be detected by the primer combination used for the SSP-PCR screening (underlined DNA sequences in Fig. 1C): AT_Charleville_ (c.1246G>C; p.Ala416Pro) [13] and AT_Cambridge II_ (c.1246G>T; p.Ala416Ser) [12], the latter by binding of the reverse primer with reduced specificity. To clarify the *SERPINC1* variants detected in the respective healthy donors, next generation sequencing was performed, revealing that these three individuals were heterozygous carriers of the point mutation c.1246G>T, described as AT_Cambridge II_ [12]. One individual gave consent to be contacted in case of relevant findings and was subsequently screened for AT antigen and activity. Both were in lower, but normal range (AT antigen 89%, AT activity 84%, with normal ranges from 80–120% for both).

### Confocal microscope analysis of GFP fusions in HEK293T cells

**Fig. 2:**
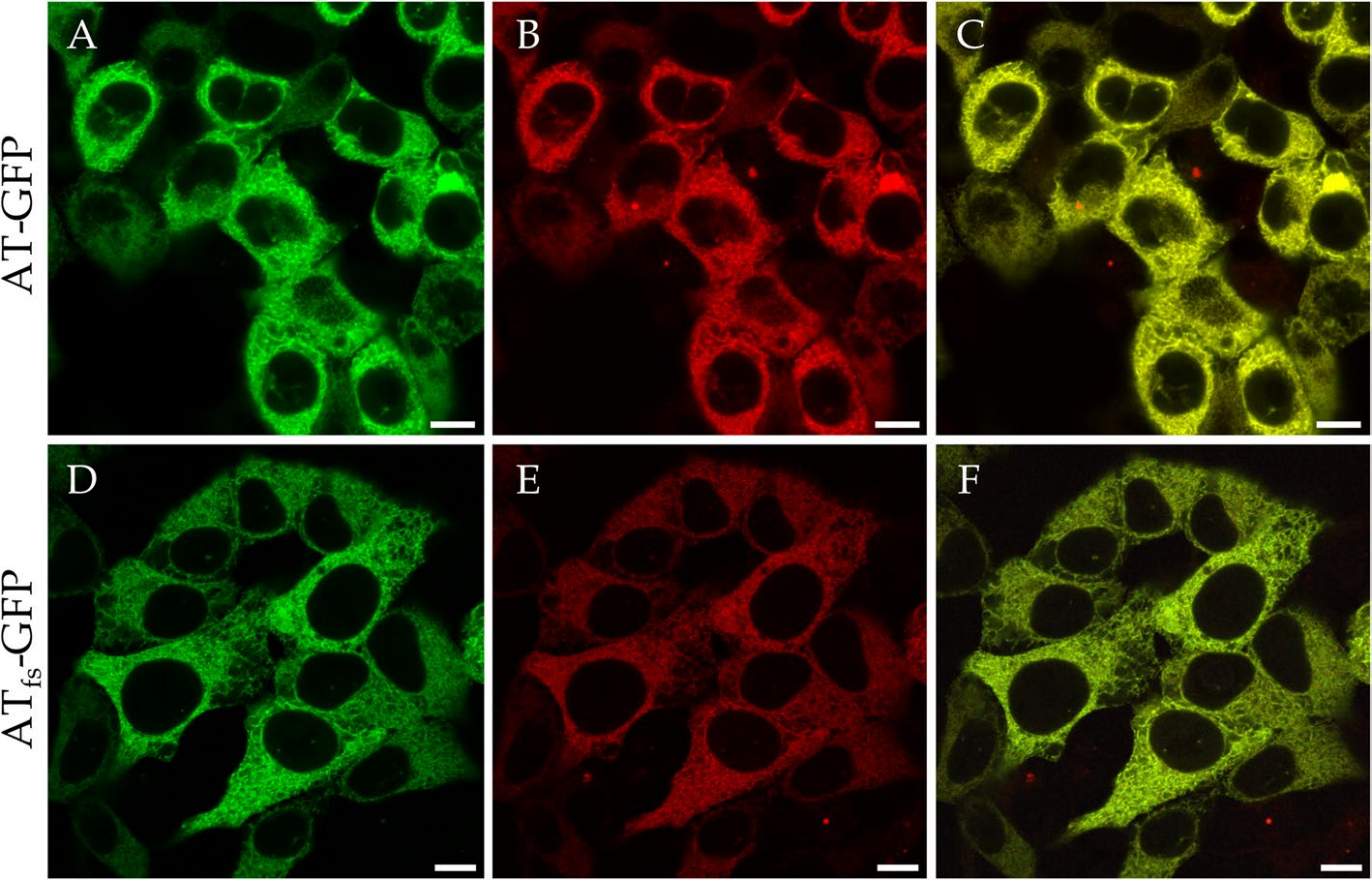
Subcellular localization of AT-GFP and AT_fs_-GFP in HEK293T cells. HEK293T cells expressing a construct encoding AT-GFP (**A**) or AT_fs_-GFP (**D**) were transfected with ER-Tracker™ Red (**B**, **E**). Both AT-GFP and AT_fs_-GFP were detected within the ER and co-localized with ER-Tracker™ Red (**C**, **F**). GFP fluorescence is shown in green, ER-Tracker™ Red in red and colocalization is indicated in yellow. Scale bars represent 10 μm

According to the type I deficiency diagnosed in our two patients, a secretory deficiency seemed likely. HEK293T cells were used as an in vitro expression system to study the secretion and subcellular localization of AT_fs_-GFP in comparison to AT-GFP. Based on the confocal laser scanning microscope analysis no apparent differences could be detected for the subcellular localization of the GFP fluorescence between *SERPINC1*-*GFP* or *SERPINC1*_c.1247dupC_-*GFP* expressing cells (Fig. 2A, D). Complete co-localization of AT-GFP and AT_fs_-GFP with ER-Tracker™ Red (Fig. 2B, C, E and F) indicated that both, AT-GFP and AT_fs_-GFP, were able to correctly enter the secretory pathway. No obvious differences concerning the structure of the endoplasmic reticulum (ER) were observed, indicating that AT_fs_ is potentially not disturbing the structural integrity of the ER (Fig. 2).

### Determination of secretion in HEK293T cells

**Fig. 3:**
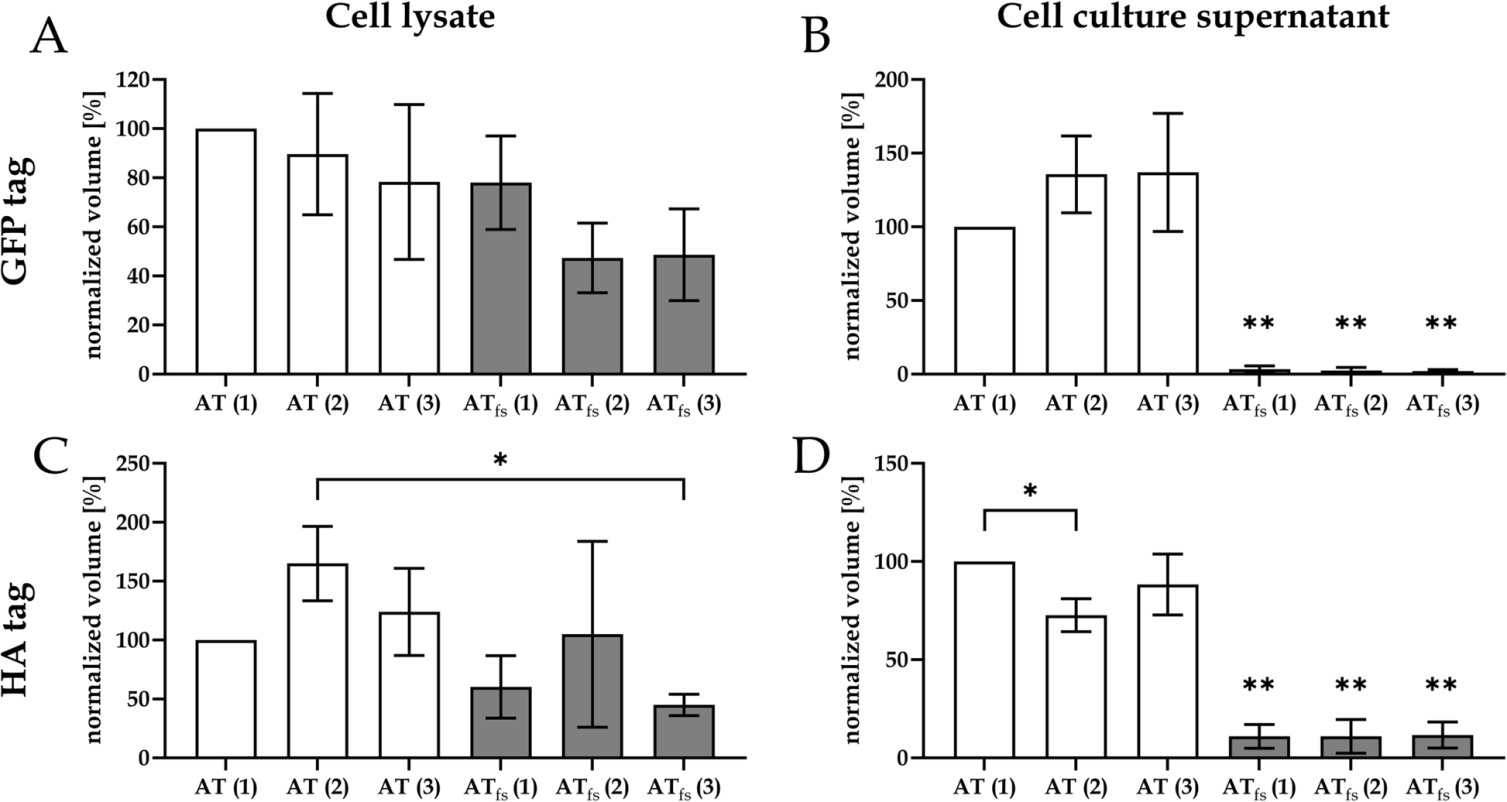
Frameshift mutation p.Ser417LysfsTer48 results in a secretion defect. Cell lysates and cell culture supernatants of HEK293T cells transfected with constructs for GFP- or HA-tagged AT or AT_fs_ were compared. While no significant quantitative differences could be detected for AT and AT_fs_ in cell lysate between all mutant and all WT lines (**A**, **C**), secretion of AT_fs_ is strongly reduced in all mutant lines compared to all WT lines with a statistical significance of *p*<0.01 (**B**, **D**) (analyzed using one-way ANOVA with Bonferroni and Holm correction, all pairs simultaneously compared). Mean values of *n*=3 technical replicates of three different cell lines per construct, error bars=standard deviation. One asterisk indicates a significant difference (*p*<0.05), two asterisks indicate a highly significant difference (*p*<0.01). If not indicated by brackets, significance refers to all mutant lines compared to all WT lines

In order to evaluate if the c.1247dupC mutation results in a secretory deficiency, supernatants and cell lysates of *SERPINC1*-*GFP* or *SERPINC1*_c.1247dupC_-*GFP* expressing HEK293T cell lines were analyzed (Fig. 3A). Three clones per construct were used for Western blot analysis, with three technical repeats per cell line. No significant difference could be observed between the amount of AT-GFP and AT_fs_-GFP within the cell lysate (Fig. 3A). Comparison of AT-GFP and AT_fs_-GFP in the cell culture supernatants showed a clear reduction in secretion of AT_fs_-GFP with a highly significant difference (*p* < 0.01) between all AT_fs_-GFP cell lines and each AT-GFP line (Fig. 3B). To exclude a potential impact of the GFP tag (which is comparably large with 239 amino acids) on the secretion of the fusion protein, constructs for fusions of AT and AT_fs_ with an HA tag (31 amino acids) were introduced into HEK293T cells. Western blot analysis was performed analogous to the analysis of GFP-tagged cell lines. Similar results as for the GFP fusions were obtained when analyzing the amount of AT-HA and AT_fs_-HA in cell lysate and cell culture supernatant (Fig. 3C, D). Supernatants of all AT_fs_-HA cells lines presented significantly reduced (*p*<0.01) secretions of the fusion protein in comparison to each AT-HA line.

These results prove a secretion defect of AT_fs_ which is in accordance with the type I AT deficiency diagnosed in our two patients.

## Discussion

We identified the frameshift mutation c.1247dupC in two patients diagnosed with type I AT deficiency. Duplication c.1247dupC results in an AT antigen with a +1 frameshift mutated aberrant C-terminus, p.Ser417LysfsTer48 (AT_fs_). The expression of recombinant constructs in HEK293T cells revealed a clear secretion defect of the AT_fs_ variant. According to literature and database research, mutation p.Ser417LysfsTer48 was so far solely mentioned in one recent publication [14], but neither indicated as a mutation identified in patients nor with an origin given for this mutation. Thus, to the best of our knowledge, we are the first to describe this mutation (i) in patients and (ii) as causative for a diagnosed AT deficiency. The *SERPINC1* c.1247dupC variant could not be identified in 358 healthy probands, thus confirming that it is not a common polymorphism.

However, three of the 358 healthy probands were found to be heterozygous carriers of AT_Cambridge II_ (c.1246G>T; p.Ala416Ser) [12]. This mutation has been described to cause transient AT deficiency (i.e., AT parameter can vary between normal and reduced values in different measurements for one and the same individual), with pathogenicity depending on environmental factors also [15]. This is in line with AT antigen levels and AT activity in a lower, but normal range und a negative family history for thromboembolic events for the one healthy donor available for further diagnostics. In addition, AT_Cambridge II_ has been described to occur with a relatively high prevalence [15, 16]. Thus, although the frequency of AT_Cambridge II_ among the analyzed healthy blood donors is surprisingly high, it is still within the realm of possibility.

A recent publication describes several AT mutants with aberrant C-termini due to +1 frameshift mutations [14]. Depending on whether the frameshift mutation occurs (a) upstream or (b) downstream of Phe440, those +1 frameshift AT variants are (a) secreted in a monomeric form or are (b) not secreted, but complexing WT AT into ER aggregates. The latter results in a disturbed ER structure, characterized by fragmentation and dilatation. This dominant-negative effect on WT AT explains, why patients harboring those frameshift variants showed more severely reduced AT activity and AT antigen levels than to be expected from a heterozygous mutation [14].

This phenomenon was not observed for the patients described here. Both had values for AT activity and AT antigen level in accordance with a standard type I AT deficiency, that is, reduction of activity and antigen amount in a range expected for a pathogenic mutation in a heterozygous state (40–60%; [14]). In line with that, secretion of AT_fs_ is strongly impaired (Fig. 3B, D), but the ER structure seems to not differ between HEK293T cells expressing *SERPINC1*-*GFP* or *SERPINC1*_c.1247dupC_-*GFP* (Fig. 2), as visualized by confocal laser scanning microscopy. Nevertheless, to eventually confirm that the integrity of the ER is in fact not impaired by the AT_fs_ mutation, further analysis by electronic microscopy would be required.

Variations from the native amino acid sequence of AT [17–19] and other SERPINS [20–22] are known to introduce conformational instability, resulting in polymerization by insertion of the reactive center loop of one mutated SERPIN molecule into the β-sheet A of a respective partner SERPIN protein, with a subsequent accumulation leading to serpinopathy [20, 23].

Mutations of a highly conserved Gly residue in strand 5B of AT (Gly456) and other members of the SERPIN family were shown to cause polymerization and intracellular retention, resulting in serpinopathy [19, 23–25]. According to the fact that not only Gly456, but actually 46 amino acids of the AT C-terminus including strands 4B and 5B are affected by the here described p.Ser417LysfsTer48 mutation, polymerization of AT_fs_ as causative for ER retention seems possible.

**Fig. 4:**
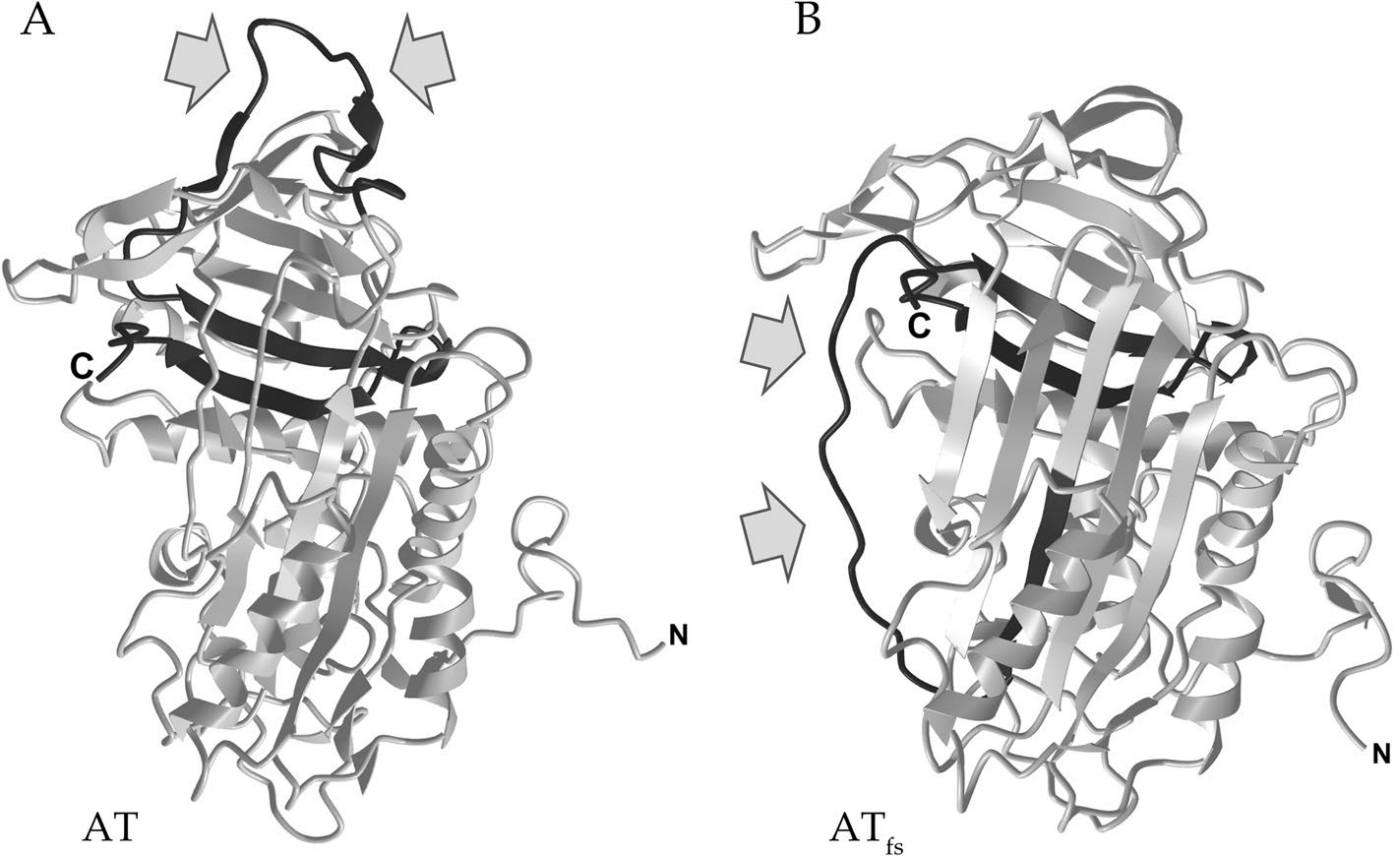
Predicted tertiary structures for WT AT and p.Ser417LysfsTer48 variant (AT_fs_). Native form of AT is indicated by the exposed reactive center loop (**A**, indicated by arrows). In silico prediction by C-I-TASSER [9] results in a folding model of AT_fs_ that resembles the latent form of SERPINs. The C-terminal peptide of AT_fs_ with altered amino acid sequence according to the frameshift mutation is differentially positioned in AT_fs_ (**B**, indicated by arrows)

Interestingly, a computer model generated with C-I-TASSER [9], comparing the (potential) tertiary structure of AT_fs_ to WT AT, shows a still SERPIN-like folding of AT_fs_ comprised of three α-helices and nine β-sheets [26] (Fig. 4B).

Since the frameshift mutation also affects the reactive center loop, the peptide at the respective position in AT_fs_ is not exposed as in native AT [26] (indicated by gray arrows in Fig. 4A), but shifted into a position mimicking the latent form of SERPIN proteins [27] (gray arrows in Fig. 4B).

The latent form of SERPINS is usually described as unable to polymerize, as the annealing site of β-sheet A is occupied and thus not available for polymerization [28].

Here, it is important to point out that algorithm-based models are known to have their limitations, as they rely on (a) the availability of high-quality structural data and (b) should consequently only be used an approximation, with recent examples highlighting structural und functional protein differences between in silico predictions and in vitro evidence [29–31]. A recent publication confirms this for five *SERPINC1* mutations resulting in AT deficiency, with conformational structures obtained from experimental evidence significantly differing from the computational predictions [32]. Thus, a possibly different conformation of AT_fs_ and hence a polymerization cannot be ruled out.

Recognition of incorrectly folded proteins is based on the presence of processed N-glycans [33], exposed hydrophobic surfaces [34, 35] or free thiols [36–38]. Structural integrity of AT is provided by three disulfide bonds (Cys40-Cys160, Cys53-Cys127, Cys279-Cys462) [39], with the C-terminal disulfide bond Cys279-Cys462 being critical for initiating a proper folding [40]. Interestingly, although frameshift mutation p.Ser417LysfsTer48 affects Cys462, a Cys at almost similar position is given by default (AT_fs_-Cys463; Fig. 1).

The predicted SERPIN-like structure of AT_fs_ may provide the necessary proximity of Cys40/Cys160, Cys53/Cys127 and Cys279/AT_fs_-Cys463 for the formation of disulfide bonds. However, the mutated C-terminus of AT_fs_ contains further Cys residues (AT_fs_-Cys420, AT_fs_-Cys421 and AT_fs_-Cys423; Fig. 1A). It was recently shown that the ER quality control for human AT exclusively relies on the thiol-dependent quality control [41]. A Cys-less AT mutant, which was shown to be misfolded and inactive, was nevertheless secreted via the conventional secretory pathway [41]. In contrast, AT variants with free thiol groups were retained in the ER [41]. Regardless of whether a potential C-terminal disulfide bond between Cys279 and AT_fs_-Cys463 is formed, there are three additional free thiol groups in AT_fs_ that will put the attention of the thiol-dependent quality control onto AT_fs_. This might explain why several other + 1 frameshift C-terminal AT variants (that do not possess additional Cys residues) are still properly secreted [14], but, in contrast, AT_fs_ is not. Failure in passing the thiol-dependent quality control might not only explain the defect in secretion, but would also be in line with the observed AT deficiency in our patients.

Thus, at least two possible mechanisms could be causative for the impaired secretion of AT_fs_ – polymerization due to aberrant folding or the presence of additional free thiol groups. Further research including conformational studies of AT_fs_ and the exchange of additional Cys residues in AT_fs_ against Ala will help to elucidate which molecular mechanism causes the secretion defect resulting in AT deficiency.

Secretion defects are often based on impaired protein folding, which leads to intervention of the ER quality control system and subsequent ER-associated protein degradation (ERAD) [34, 42–44]. The use of proteasome inhibitors emphasized the degradation of different AT mutants [45, 46] and other mutated proteins from the SERPIN family [47–51] via ERAD and might also elucidate the probably ERAD-dependent degradation of AT_fs_.

AT deficiency and, moreover, combined defects such as AT deficiency and FVL, as reported in our patients, are a strong risk factor for thrombotic events. A meta-analysis of 19 studies reports an estimated odds ratio of 14.0 for venous thromboembolism in patients with AT deficiency [52] while another meta-analysis, summarizing eight studies, reports an odds ratio of 4.17 for FVL [53]. Comparably, the annual incidence for thrombosis is 1.7% for individuals with AT deficiency and 0.1–0.2% for patients with FVL (with 0.01% in the overall population without identified genetic predisposition) [5]. Moreover, patients with AT deficiency are younger on average (around 40 years) when their first thrombotic event occurs than those with a heterozygous FVL mutation (around 60 years) [54]. Therefore, since the AT deficiency is a strong risk factor and because the patients suffered their first thromboembolic event at a young age, it is more likely that the AT deficiency had greater impact on the thrombotic events in the patients.

## Conclusion

Here, we describe for the first time mutation c.1247dupC (p.Ser417LysfsTer48) as causative in patients suffering from type I AT deficiency. A clear defect in secretion of the GFP- or HA-tagged AT_fs_ was shown in a HEK293T in vitro expression system. Impaired secretion of AT_fs_ might be caused by polymerization induced by aberrant folding or by new Cys residues introduced by the frameshift mutation, providing free thiol groups that are recognized by the thiol-dependent ER quality control.

### Supplementary Information

**Additional file 1: Supplemental Figure 1.** Screening of healthy donors 1-93 for WT allele. **Supplemental Figure 2.** Screening of healthy donors 1-93 for mutant allele.** Supplemental Figure 3. **Screening of healthy donors 94-186 for WT allele.** Supplemental Figure 4. **Screening of healthy donors 94-186 for mutant allele.** Supplemental Figure 5.** Screening of healthy donors 187-279 for WT allele.** Supplemental Figure 6.** Screening of healthy donors 187-279 for mutant allele.** Supplemental Figure 7. **Screening of healthy donors 280-360 for WT allele.** Supplemental Figure 8. **Screening of healthy donors 280-360 for mutant allele.** Supplemental Figure 9.** Repetition of PCRs with no or positive results in first PCR.

## Data Availability

The datasets used and analyzed during the current study are available from the corresponding author on reasonable request.

## Abbreviations

AT: Antithrombin
AT_fs_: Antithrombin with p.Ser417LysfsTer48 mutation
CDS: Coding sequence
DVT: Deep vein thrombosis
ER: Endoplasmic Reticulum
ERAD: Endoplasmic Reticulum-associated protein degradation
FVL: Factor V Leiden
GFP: Green fluorescent protein
HA: Hemagglutinin
SERPIN: Serine protease inhibitor
SSP-PCR: Sequence specific primer-polymerase chain reaction
