# Supplementary material for: SERPINC1 c.1247dupC: a novel SERPINC1 gene mutation associated with familial thrombosis results in a secretion defect and quantitative antithrombin deficiency
Source: Thromb J. 2024 Feb 12;22:19. doi: 10.1186/s12959-024-00589-5 (PMC10860291; doi:10.1186/s12959-024-00589-5)
Supplement: Supplementary file 1 — Additional file 1: Supplemental Figure 1. Screening of healthy donors 1-93 for WT allele. Supplemental Figure 2. Screening of healthy donors 1-93 for mutant allele. Supplemental Figure 3. Screening of healthy donors 94-186 for WT allele. Supplemental Figure 4. Screening of healthy donors 94-186 for mutant allele. Supplemental Figure 5. Screening of healthy donors 187-279 for WT allele. Supplemental Figure 6. Screening of healthy donors 187-279 for mutant allele. Supplemental Figure 7. Screening of healthy donors 280-360 for WT allele. Supplemental Figure 8. Screening of healthy donors 280-360 for mutant allele. Supplemental Figure 9. Repetition of PCRs with no or positive results in first PCR. [file 12959_2024_589_MOESM1_ESM.pdf]

Supplemental Material: Uncropped gel images of SSP-PCR screening for the *SERPINC1* c.1247dupC mutation in healthy individuals.

Genomic DNAs of 360 healthy blood donors were used for screening (indicated as "healthy donors" with a respective numbering in the subsequent figure legends). Genomic DNA of a patient described in this study was used as positive control (indicated as "patient described") for the mutant allele. Genomic DNA of a proband from another study whose *SERPINC1* gene was analyzed by next generation sequencing and proven without pathogenic *SERPINC1* mutation was used as negative control (indicated as "healthy control") for the mutant allele. Two healthy donors were excluded from final counting of individuals tested due to insufficient amounts of genomic DNA, resulting in 358 healthy individuals tested.

All individuals were tested for *SERPINC1* wild-type (WT) c.1247C allele and mutant c.1247dupC allele.

For all images, lanes are counted from top row to bottom row, left to right, with small lanes used for DNA ladder counting as first lane per row.

Ladder fragments in each gel are top to bottom 1000 bp, 500 bp, 400 bp, 300 bp, 200 bp, 100 bp and 50 bp.

Supplemental Figure 1: Screening of healthy donors 1-93 for WT allele.

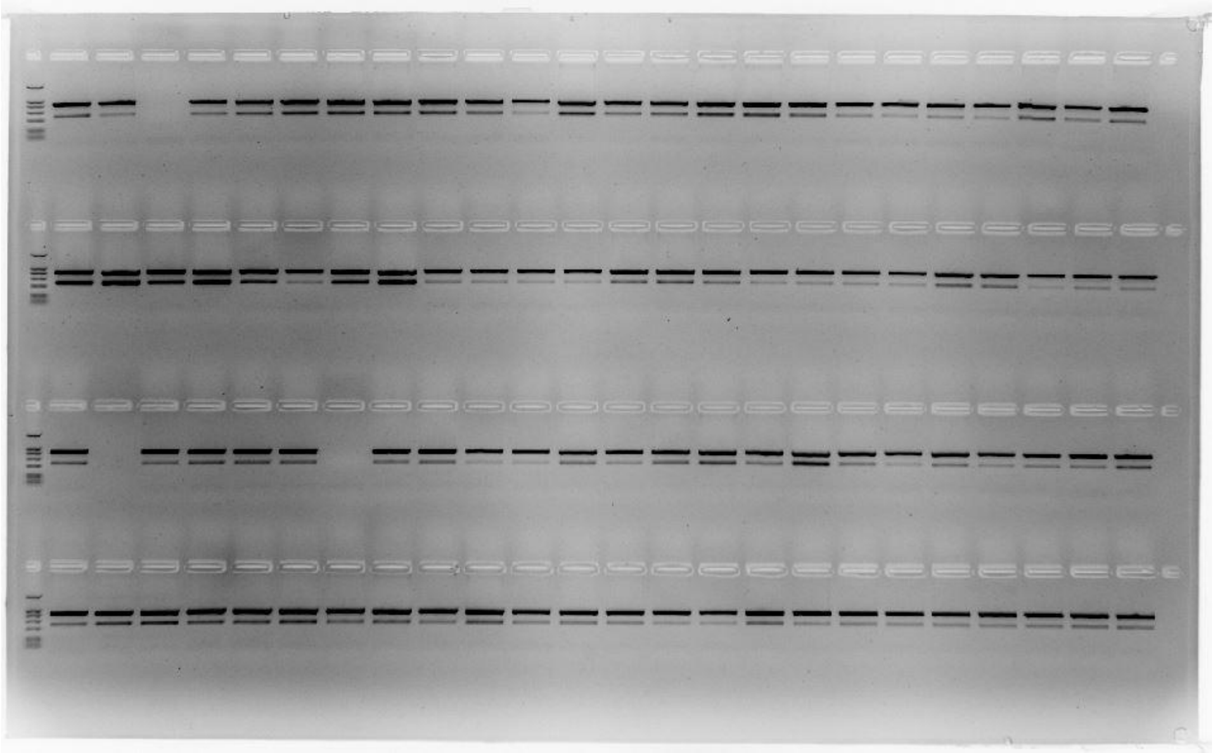

First row: Ladder (lane 1), PCRs for healthy control (lane 2), patient described (lane 3), water control (lane 4), healthy donor samples 1-21 (lanes 5-25).

Second row: Ladder (lane 1), PCRs for healthy donors 22-45 (lanes 2-25).

Third row: Ladder (lane 1), PCRs for healthy donors 46-69 (lanes 2-25).

Fourth row: Ladder (lane 1), PCRs for healthy donors 70-93 (lanes 2-25).

The PCR result for the *SERPINC1* WT allele is represented by a 238 bp fragment. A 434 bp fragment from *GH1* gene was used as internal PCR control.

Healthy blood donors 47 and 52 were excluded from final counting of blood donors tested because of limited DNA amount.

Lanes 1-4 from first row were used as image for Figure 1B (upper left image), with lane 2 designated as C, lane 3 as P and lane 4 as H_2O.

Supplemental Figure 2: Screening of healthy donors 1-93 for mutant allele.

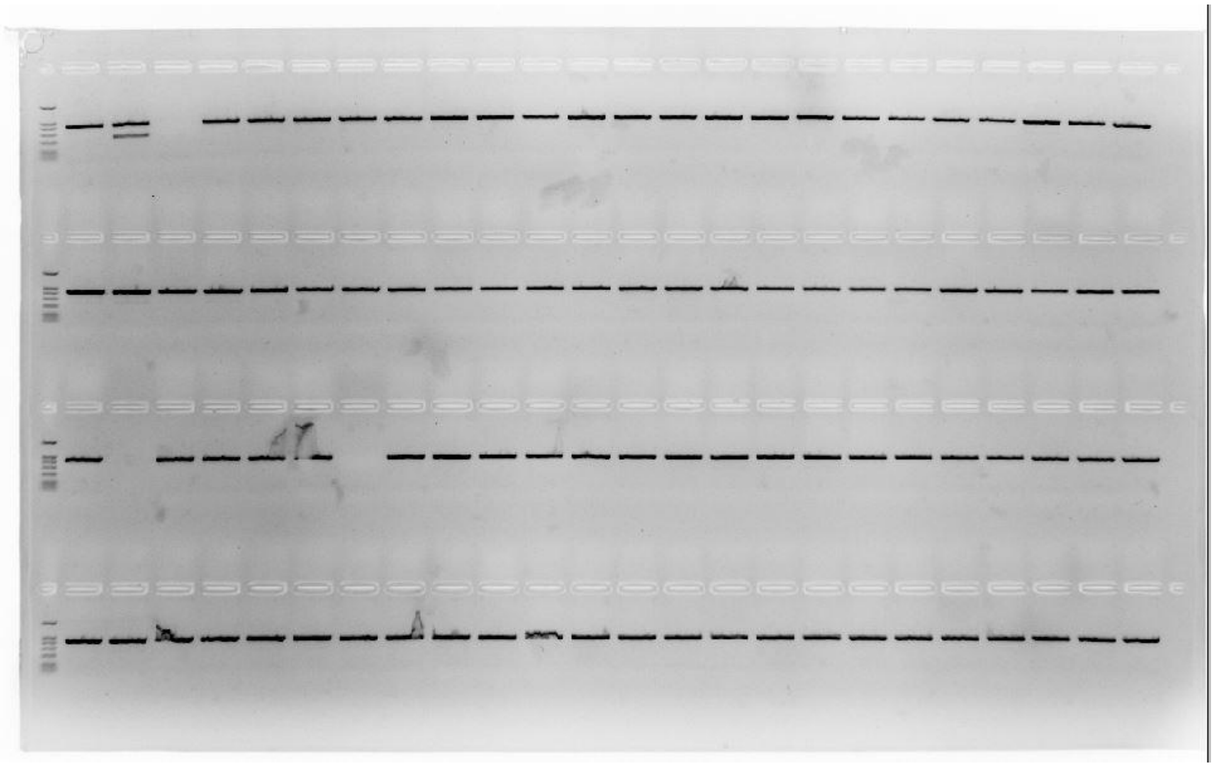

First row: Ladder (lane 1), PCRs for healthy control (lane 2), patient described (lane 3), water control (lane 4), healthy donors 1-21 (lanes 5-25).

Second row: Ladder (lane 1), PCRs for healthy donors 22-45 (lanes 2-25).

Third row: Ladder (lane 1), PCRs for healthy donors 46-69 (lanes 2-25).

Fourth row: Ladder (lane 1), PCRs for healthy donors 70-93 (lanes 2-25).

The PCR result testing for the *SERPINC1* c.1247dupC mutation is represented by a 239 bp fragment. A 434 bp fragment from *GH1* gene was used as internal PCR control. Healthy blood donors 47 and 52 were excluded from final counting of blood donors tested because of limited DNA amount.

Supplemental Figure 3: Screening of healthy donors 94-186 for WT allele.

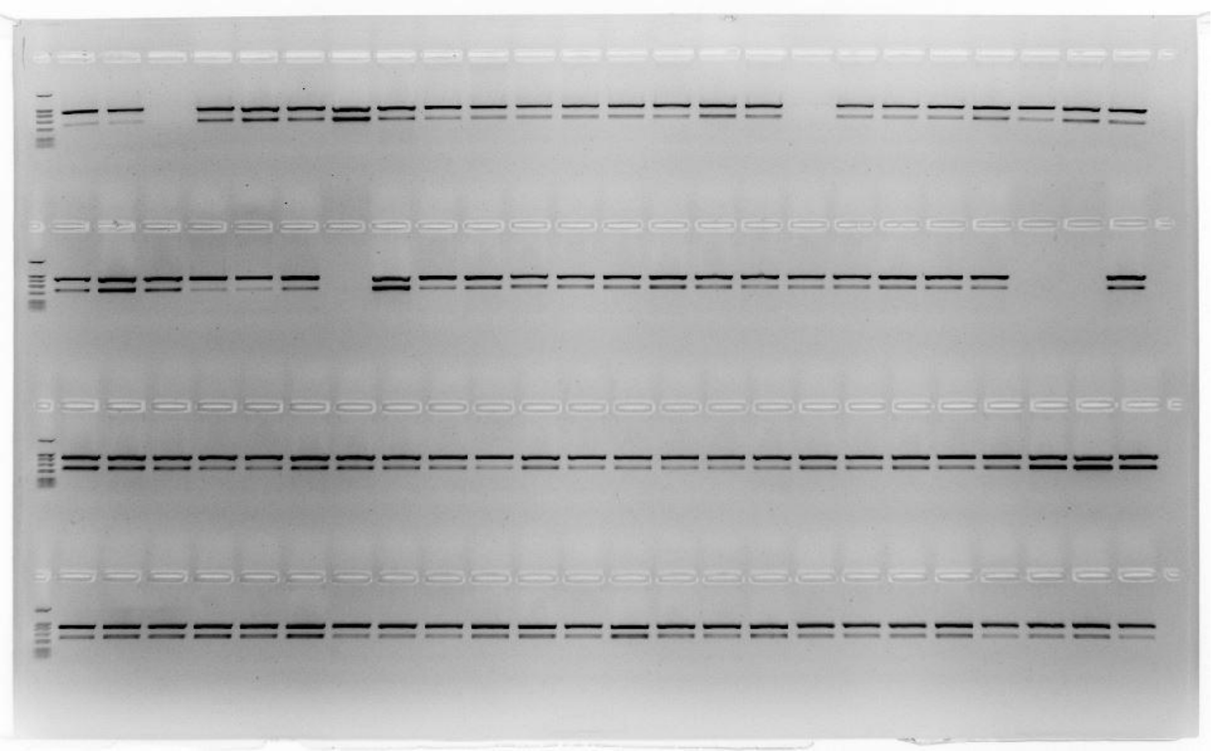

First row: Ladder (lane 1), PCRs for healthy control (lane 2), patient described (lane 3), water control (lane 4), healthy donors 94-114 (lanes 5-25).

Second row: Ladder (lane 1), PCRs for healthy donors 115-138 (lanes 2-25).

Third row: Ladder (lane 1), PCRs for healthy donors 139-162 (lanes 2-25).

Fourth row: Ladder (lane 1), PCRs for healthy donors 163-186 (lanes 2-25).

The PCR result for the *SERPINC1* WT allele is represented by a 238 bp fragment. A 434 bp fragment from *GH1* gene was used as internal PCR control.

PCRs with no result (107, 121, 136, 137) were repeated separately (see Supplemental Figure 9).

Supplemental Figure 4: Screening of healthy donors 94-186 for mutant allele.

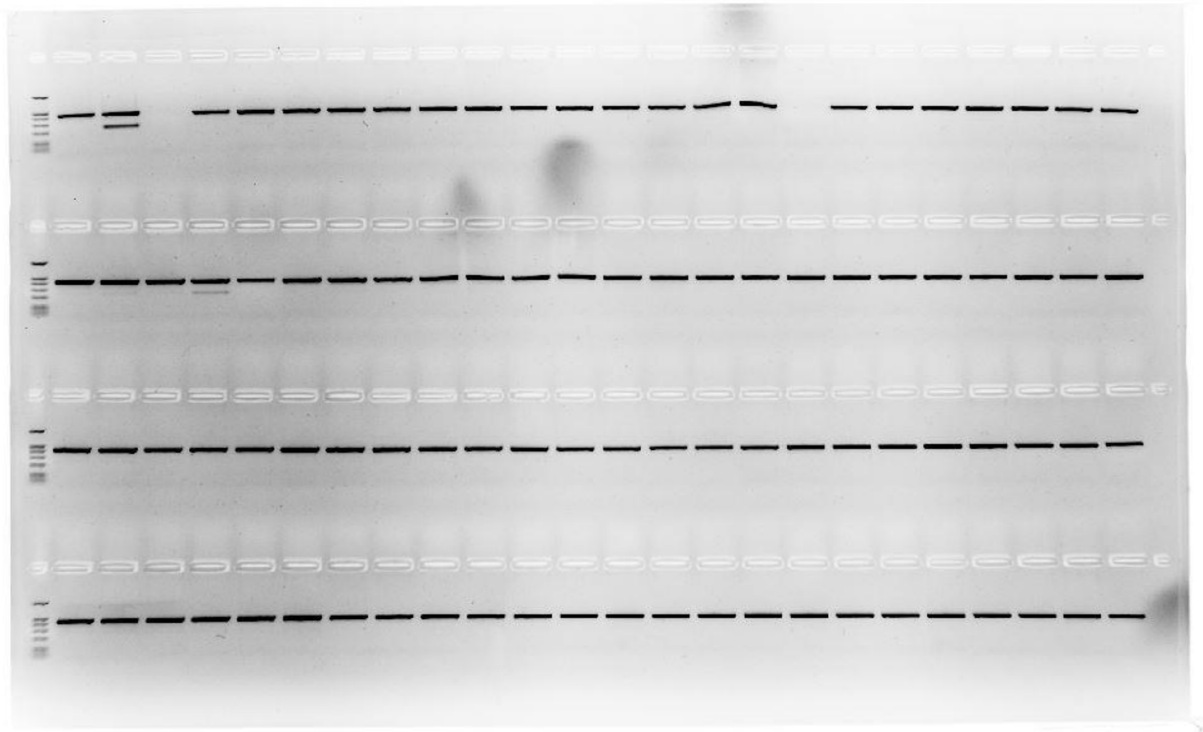

First row: Ladder (lane 1), PCRs for healthy control (lane 2), patient described (lane 3), water control (lane 4), healthy donors 94-114 (lanes 5-25).

Second row: Ladder (lane 1), PCRs for healthy donors 115-138 (lanes 2-25).

Third row: Ladder (lane 1), PCRs for healthy donors 139-162 (lanes 2-25).

Fourth row: Ladder (lane 1), PCRs for healthy donors 163-186 (lanes 2-25).

The PCR result testing for the *SERPINC1* c.1247dupC mutation is represented by a 239 bp fragment. A 434 bp fragment from *GH1* gene was used as internal PCR control. PCRs with no (107) or positive result (118) were repeated separately (see Supplemental Figure 9).

Supplemental Figure 5: Screening of healthy donors 187-279 for WT allele.

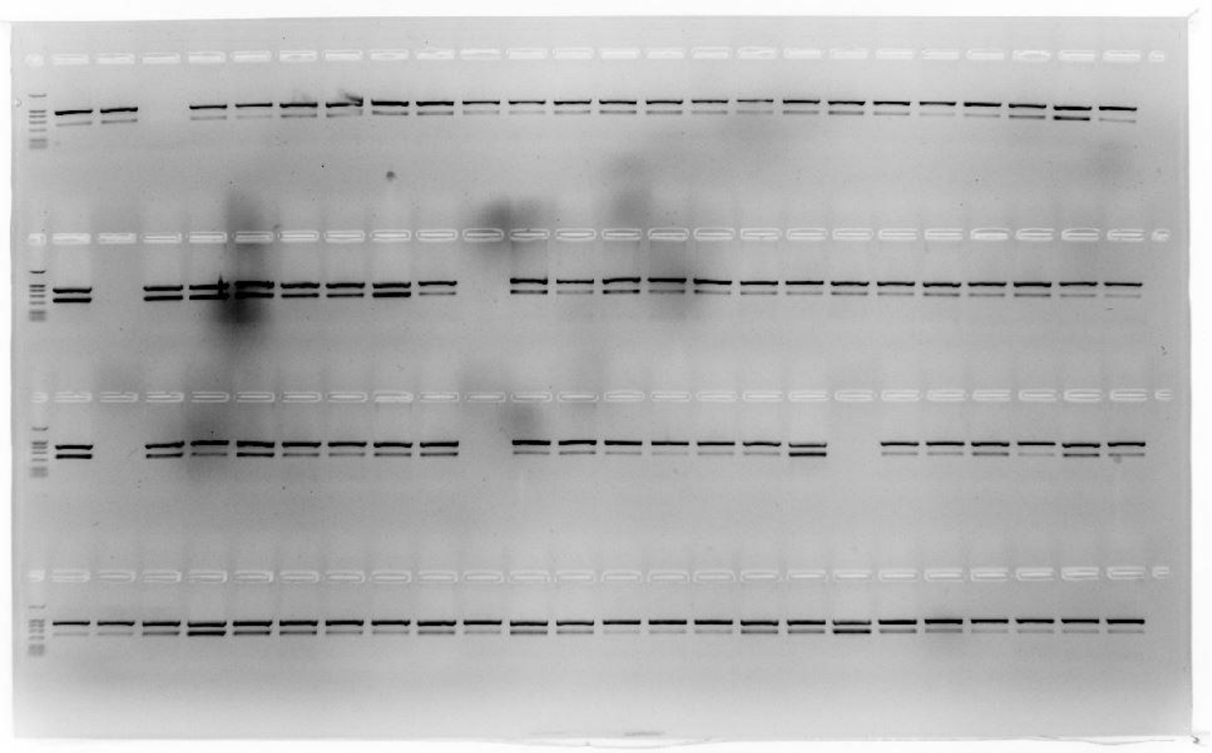

First row: Ladder (lane 1), PCRs for healthy control (lane 2), patient described (lane 3), water control (lane 4), healthy donors 187-207 (lanes 5-25).

Second row: Ladder (lane 1), PCRs for healthy donors 208-231 (lanes 2-25).

Third row: Ladder (lane 1), PCRs for healthy donors 232-255 (lanes 2-25).

Fourth row: Ladder (lane 1), PCRs for healthy donors 256-279 (lanes 2-25).

The PCR result for the *SERPINC1* WT allele is represented by a 238 bp fragment. A 434 bp fragment from *GH1* gene was used as internal PCR control.

PCRs with no result (209, 217, 233, 241, 249) were repeated separately (see Supplemental Figure 9).

Lanes 7-9 from second row were used as image for Figure 1B (upper right image), with lane 7 designated as H1, lane 8 as H2 and lane 9 as H3.

Supplemental Figure 6: Screening of healthy donors 187-279 for mutant allele.

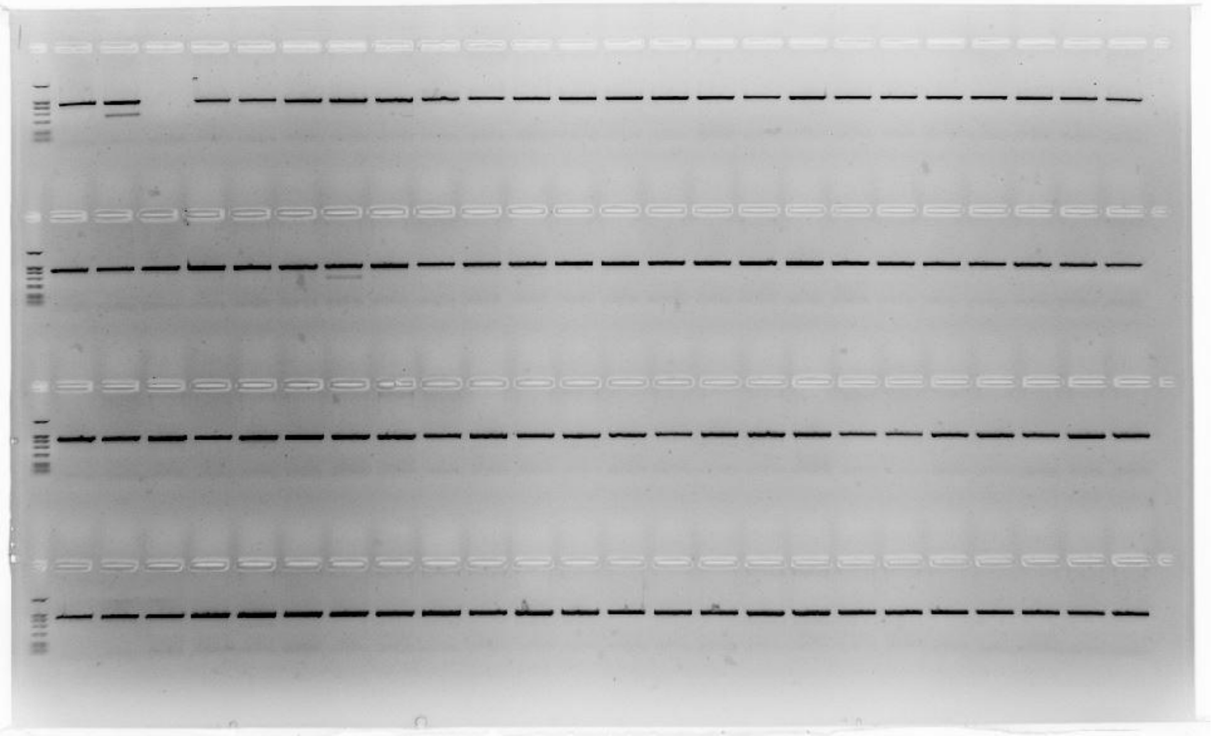

First row: Ladder (lane 1), PCRs for healthy control (lane 2), patient described (lane 3), water control (lane 4), healthy donors 187-207 (lanes 5-25).

Second row: Ladder (lane 1), PCRs for healthy donors 208-231 (lanes 2-25).

Third row: Ladder (lane 1), PCRs for healthy donors 232-255 (lanes 2-25).

Fourth row: Ladder (lane 1), PCRs for healthy donors 256-279 (lanes 2-25).

The PCR result testing for the *SERPINC1* c.1247dupC mutation is represented by a 239 bp fragment.

PCR with positive result (214) was repeated separately (see Supplemental Figure 9).

Lanes 1-4 from first row and lanes 7-9 from second row were used as image detail for Figure 1B (lower left and lower right image), with lane 2 designated as C, lane 3 as P, lane 4 as H_2O, lane 7 designated as H1, lane 8 as H2 and lane 9 as H3.

Supplemental Figure 7: Screening of healthy donors 280-360 for WT allele.

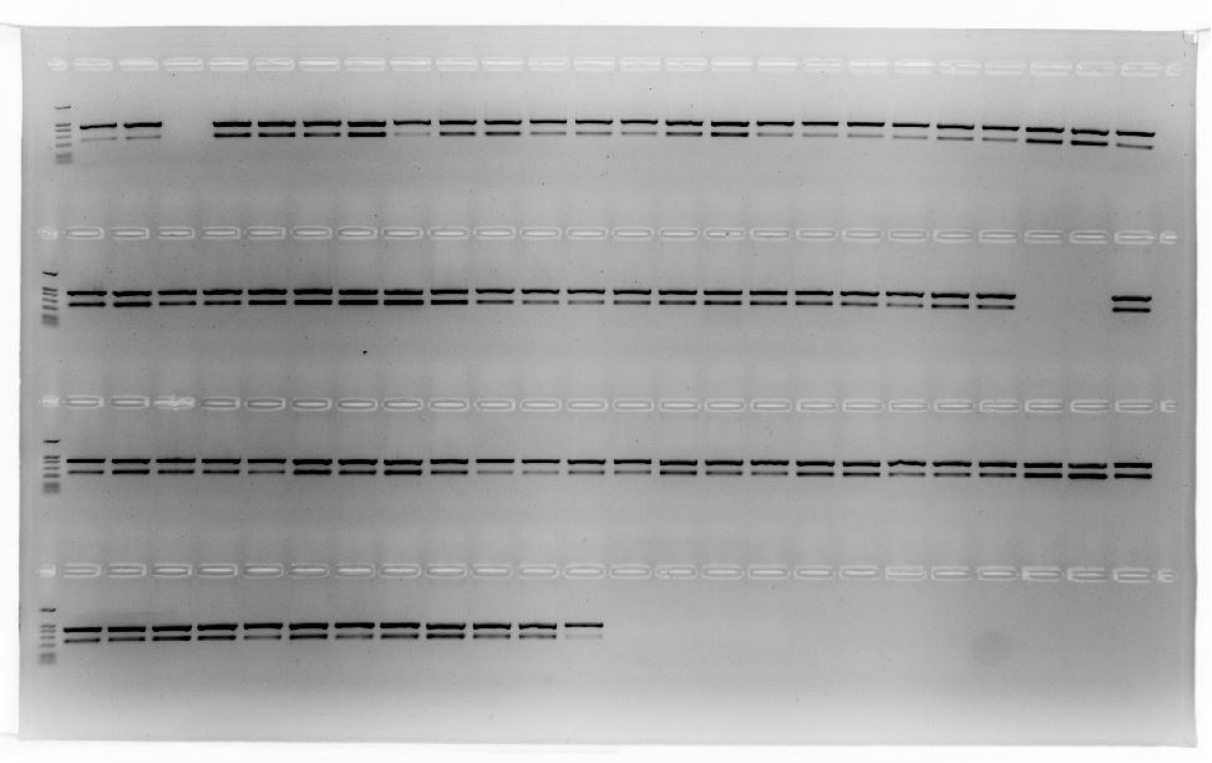

First row: Ladder (lane 1), PCRs for healthy control (lane 2), patient described (lane 3), water control (lane 4), healthy donors 280-300 (lanes 5-25).

Second row: Ladder (lane 1), PCRs for healthy donors 301-324 (lanes 2-25).

Third row: Ladder (lane 1), PCRs for healthy donors 325-348 (lanes 2-25).

Fourth row: Ladder (lane 1), PCRs for healthy donors 349-360 (lanes 2-13).

The PCR result for the *SERPINC1* WT allele is represented by a 238 bp fragment. A 434 bp fragment from *GH1* gene was used as internal PCR control.

PCRs with no result (322, 323) were repeated separately (see Supplemental Figure 9).

Supplemental Figure 8: Screening of healthy donors 280-360 for mutant allele.

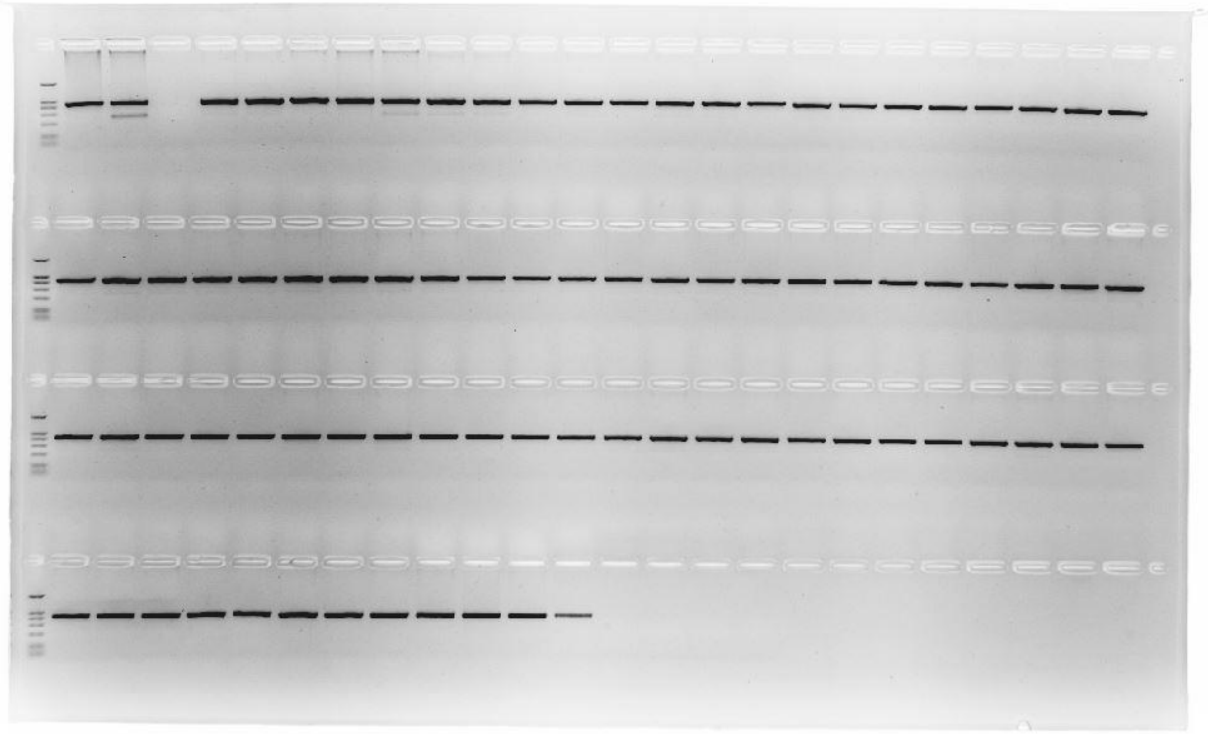

First row: Ladder (lane 1), PCRs for healthy control (lane 2), patient described (lane 3), water control (lane 4), healthy donors 280-300 (lanes 5-25).

Second row: Ladder (lane 1), PCRs for healthy donors 301-324 (lanes 2-25).

Third row: Ladder (lane 1), PCRs for healthy donors 325-348 (lanes 2-25).

Fourth row: Ladder (lane 1), PCRs for healthy donors 349-360 (lanes 2-13).

The PCR result testing for the *SERPINC1* c.1247dupC mutation is represented by a 239 bp fragment. A 434 bp fragment from *GH1* gene was used as internal PCR control. PCR with positive result (284) was repeated separately (see Supplemental Figure 9).

Supplemental Figure 9: Repetition of PCRs with no or positive results in first PCR.

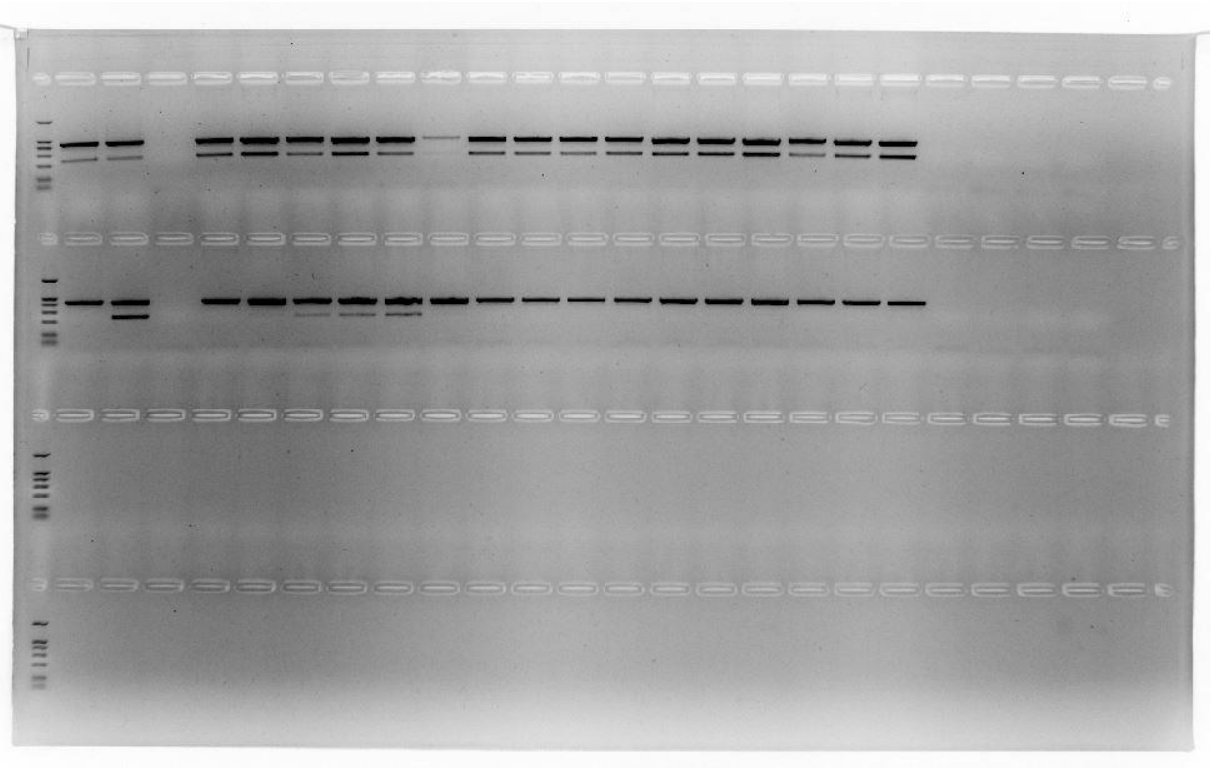

First row (screening for WT allele) and second row (screening for mutant allele): Ladder (lane 1), PCRs for healthy control (lane 2), patient described (lane 3), water control (lane 4), healthy donors 10, 62 (both random control), 118, 214, 284 (positive for mutant allele in first PCR), 107, 121, 136, 137, 209, 217, 233, 241, 249, 322, 323 (no result in first PCR) (lanes 5-20).

Third and fourth row: Ladder (lane 1).

The PCR result for the *SERPINC1* WT allele (first row) is represented by a 238 bp fragment. The PCR result testing for the *SERPINC1* c.1247dupC mutation (second row) is represented by a 239 bp fragment. A 434 bp fragment from *GH1* gene was used as internal PCR control.

Positive PCR results for the mutant allele were confirmed for healthy blood donors 118, 214 and 284.
